# Risk Factors for Myopia: A Review

**DOI:** 10.3390/jcm12186062

**Published:** 2023-09-19

**Authors:** Noelia Martínez-Albert, Inmaculada Bueno-Gimeno, Andrés Gené-Sampedro

**Affiliations:** 1 Avanza Ophthalmology Clinic, 46007 Valencia, Spain; noelia.martinez@uv.es; 2 Department of Optics and Optometry and Vision Sciences, University of Valencia, 46100 Burjassot, Spain; inmaculada.bueno@uv.es; 3 Research Institute on Traffic and Road Safety (INTRAS), University of Valencia, 46022 Valencia, Spain

**Keywords:** myopia, incidence, risk factors, near work, time outdoors, intervention

## Abstract

Due to the myopia prevalence increase worldwide, this study aims to establish the most relevant risk factors associated with its development and progression. A review search was carried out using PubMed, Web of Science, and Scopus databases to identify the main myopia risk factors. The inclusion criteria for the articles were those related to the topic, carried out in subjects from 5 to 30 years, published between January 2000 and May 2023, in English, and with the full text available. Myopia etiology has proven to be associated with both genetic and environmental factors as well as with gene–environment interaction. The risk of developing myopia increases in children with myopic parents (one parent ×2 times, two parents ×5 times). Regarding environmental factors, education is the main risk factor correlated with myopia prevalence increase. Further, several studies found that shorter distance (<30 cm) and longer time spent (>30 min) for near work increase the risk of myopia. Meanwhile, increased outdoor activity (>40 min/day) has been shown to be a key factor in reducing myopia incidence. In conclusion, the interventional strategy suggested so far to reduce myopia incidence is an increase in time outdoors and a reduction in the time spent performing near-work tasks.

## 1. Introduction

Myopia is one of the most common refractive disorders, which occurs when there is no harmony between ocular power and eye length growth. Because of this decompensation, the image of distant objects results in being out of focus. Distance objects are focused anteriorly to the retinal plane, resulting in a blurry perception of the visual images. Even though the refractive error changes from birth to adulthood, the major changes occur during childhood when the eye develops. The emmetropization mechanism is responsible for the compensation between the changes of the ocular components during eye development to guide the refractive error towards emmetropia [1]. The ametropia presence in adulthood is considered the result of the failure of emmetropization or emmetropia maintenance.

The prevalence of myopia is globally growing, and nearly half of the world’s population may be myopic by 2050, with around 10% highly myopic [2]. Myopia has reached epidemic levels in the developed countries of East and Southeast Asia. In these regions, the prevalence has been increasing over time and especially at younger ages. Increased incidence has been reported in populations from 6 years of age [3,4], and the prevalence has reached values between 80–90% in teenagers [5]. Similarly, Europe has undergone an increment of myopia prevalence through the years [6]. Prevalence increases in European children from 7–8 years of age [7], and the reported prevalence ranges from 17 to 36% in teenagers with European ancestry [8,9]. Further, myopia prevalence has been demonstrated to be greater among the population with the highest education. Regions with greater expansion of urbanization and higher education have shown an earlier increase in both Europe [6,8,10] and Asia [10]. Thus, myopia prevalence is only between 5–10% for young adults in less developed regions because of the lower educational evolution [11].

High myopia has also increased as a result of the increased myopia prevalence in younger people as well as the rates of progression [9]. In Spain, Alvarez-Peregrina et al. [12] have recently reported an increment in high myopia from 1.7% to 3.6% in children between 5 and 7 years over one year. Previous studies [13,14] have demonstrated that the later the age of onset is, the lower the risk of progressing to high myopia, since an earlier age allows myopia more time to progress. Hence, myopia has become a public health issue since it is currently one of the causes of significant visual loss [15]. This is due to its associated complications, which turn it into pathological myopia. Fricke et al. [16] have estimated the myopic maculopathy prevalence to be 0.57% of the global population by 2050 if actions are not taken to manage the development and progression of myopia. Myopia has also been shown as a risk of suffering some ocular conditions such as retinal detachment [17], glaucoma [18], and cataracts [19].

Nowadays, myopia development and progression are not completely understood. It is known that both genetic and environmental factors play a role in its etiology. Risk factors for young-onset myopia can include a combination of genetic, environmental, and lifestyle factors. Previous reviews [11,20,21,22] have reported genetics, near work, and time outdoors as risk factors for myopia development. However, some of these risk factors are interrelated or linked to other factors. There is still a need to identify those risk factors that can be modifiable to provide interventions during childhood. Thus, this review aimed to discuss the principal risk factors for myopia development and the interconnections between them. In this way, this work showed the key factors that are associated with an increased risk of developing myopia at a young age.

## 2. Methodology

PubMed, Web of Science, and Scopus databases were used to perform the review search using a combination of the following keywords: myopia, risk factors, review, and etiology. Once the principal risk factors were identified, the search was performed again using the combination of myopia with other keywords such as “parental”, “genetic” “near work”, “education”, “time outdoors”, “light exposure”, and “environment”, among others. Moreover, the reference list of the available articles was also reviewed. 

The literature obtained was first screened by title and in order to choose the articles related to the topic of this review. Then, the full-text articles were evaluated for their inclusion. Eligibility criteria are shown in Table 1. 

From the different databases, 4607 records were obtained in the search, from which 2530 duplicates were removed. Then, the articles were first screened by topic, and 1771 reports were removed. From a total of 759 articles, 227 were included according to the eligibility criteria (Table 1). Finally, 122 articles were removed because there was an overlapping topic or irrelevant data. Thus, 105 articles were included in the results of the present review.

## 3. Results

Myopia is complex in etiology as both genetic and environmental factors are involved in its development and progression as well as the gene–environment interaction may have an important role. Table 2 summarizes the main risk factors for myopia at the end of this section.

### 3.1. Genetic Factors

The genetic contribution has been evidenced by familiar and genome-wide association studies. Previous studies have already shown children who have myopic parents are highly likely to become myopic compared with those who do not [23,24,25,26]. Ip et al. [26] reported that myopia prevalence increases in children with the number of myopic parents, from 14.9% for one myopic parent to 43.6% for two myopic parents. Similarly, Jones et al. [27] informed that two myopic parents raised the risk of having myopia 5.07-fold, and one parent raised it 2.08-fold. Nonetheless, the relation of refractive error between children and parents may be partially due to families sharing the environment in addition to genes. Studies in monozygotic twins have provided a better understanding of myopia heritability, as they have the same genes and share a similar environment. The findings of these studies have exhibited that monozygotic twins have more similar refractive error and ocular components than dizygotic [28,29,30,31]. 

Recently, one genome-wide association meta-analysis has established 161 independent loci for refractive error [32]. Genome-wide association studies have pointed out myopia polygenicity, even though the current findings only account for up to 10% of the refractive error [32,33,34]. Additionally, there is evidence of the environmental influence on phenotypic variation. The gene–environment interaction for refractive error has been assessed to look if the response of the different genotypes might be different in the same environment. That is to say, whether some genotypes are more susceptible to changes than others in the same environment. Studies in adults have evidenced that educational level influences the genetic risk of myopia development [22,35,36]. 

There are numerous syndromes to which high myopia is related, such as Marfan, Knobloch, or congenital stationary night blindness, although these myopia forms are only present in a few percent of the population. These syndromes are linked to genetic mutations affecting the connective tissue [37,38,39]. Similarly, non-syndromic high myopia has been associated with some chromosomal locations and candidate genes [40,41,42]. However, these findings cannot explain all cases of either inherited syndromic or non-syndromic high myopia.

### 3.2. Environmental Factors

The rise of myopia prevalence, particularly in some regions, does not seem to be due to only genetic heritage, being that genes cannot change in such a rapid way. Actually, populations with the same ethnic background have exhibited different myopia prevalence depending on the environment they live in [43,44,45].

#### 3.2.1. Education and Near Work

Educational level has a strong correlation with myopia prevalence, which agrees with the fact that myopia progresses during the school years. Higher education level has been associated with higher myopia prevalence throughout different populations in both Europe and Asia [6,8,10,46,47]. Instead, regions with lower educational evolution have shown low myopia prevalence, below 10% [11]. The changes in educational policies have been suggested to have an impact on myopia prevalence rise in East and Southeast Asia [11,48,49,50]. In China, myopia prevalence has changed over the years, reaching 80% in young adults (17–29 years) because of an increment in higher education enrolment and changes to stricter criteria for access based on academic performance [48,49]. Further, both years and intensity of study should be taken into account to evaluate the effect of education on myopia. The increase in mandatory school years has proved to be related to myopia prevalence increment in the United Kingdom [51]. Higher school performance [24,52] and intelligence quotient [25,53] have also been shown to be related to myopia in children from 7 to 13 years. Aligned with that, attending extra tuition classes increased the risk of myopia incidence in children (7–15 years) and young adults (18–23 years) [10,54,55]. Therefore, changes in the educational systems, considering school length and performance intensity, have been demonstrated to be associated with the rise in myopia prevalence [11,48,49,50,51].

Near work might be considered involved in such a correlation between education and myopia. Study styles, which involve near-work activities, are assumed to have an influence on myopia development [56]. Several studies [57,58,59,60,61,62] have acquired an association of near work with both myopia development and progression in children (7–15 years). This association was stronger in younger children in two studies, and therefore, with earlier onset [57,58]. Guo et al. [59] found that both shorter distance and longer time spent for near work increase the risk of myopia in children aged between 12–15 years. A 22-year follow-up [63] in schoolchildren (8–13 years) obtained an association of adulthood high myopia (spherical equivalent ≤ −6.00 D) with more time spent on reading and close work. Contrarily, other authors have reported no correlation between near work and myopia incidence [25,27,64] nor its progression [65,66] in samples of children aged from 6 to 15 years. Meanwhile, Jones-Jordan et al. [67] did not find evidence of the relationship between near work and myopia development in children from 6 to 14 years since the visual activity became different once the myopia was onset. A recent meta-analysis [20] stated that each diopter hour of near work per week leads to 2% increased odds of having myopia based on articles than involved children. Other authors have found a relationship between reading and myopia rather than with near work per se [57,62,68,69]. Moreover, the Sydney myopia study [62] reported continuous reading (>30 min) and closer reading distance (<30 cm) to increase the odds of having myopia in children (12–13 years) by 1.5 and 2.5, respectively. This showed up the relevance of near-work intensity as a risk factor. Faster myopic progression has been linked with shorter reading distance (<25 cm) [61] and more near-work time [60] in children (6–17 years). Scheiman et al. [70] suggested a relationship between near-work activity and myopia stabilization in children (6–11 years). Concretely, each additional hour of near work per week would decrease the odds of myopia stabilization by 2% at 15 years old. 

Traditionally, the greater accommodation in use has been thought to be the link between near work and myopia. Myopic children (6–18 years) have exhibited significantly less accommodative response than emmetropic [71]. Other authors reported higher variability of the accommodative response in myopes [72,73]. Nevertheless, animal studies have elucidated that even when accommodation is inactive, the eye growth remains working [74,75,76]. Likewise, the findings of animal models have raised the hypothesis that the hyperopic defocus produced as a result of accommodative lag may influence myopia development [77,78]. Greater accommodative lag in myopic children has been found in some studies [79,80]. Mutti et al. [81] reported that increased lag of accommodation is observed the year after myopia onset in children between 6 and 15 years. However, longitudinal studies reported no correlation between accommodative lag and myopia progression in children from 6 to 15 years [82,83,84].

#### 3.2.2. Time Outdoors and Light Exposure

At first, some studies associated less time in sports activity as a risk factor for myopia [24,27]. Further studies obtained that lower myopia was associated with higher time spent outdoors rather than time of sports practice [58,62,64,66,85,86,87]. This fact suggests a greater time spent outdoors might protect from myopia development. Instead, a few authors informed of no influence of outdoor activity on myopia development [25,88,89].

The Avon Longitudinal Study of Parents and Children [90] reported that the additional time spent outdoors in children between 3 and 9 years reduced myopia incidence at the age of 10 and 15 years. Hence, the longitudinal study of Pärssinen and Kauppinen [63] obtained that a lower time of outdoor activities was associated with adulthood high myopia. Previous studies [27,85] even found that the influence of parental myopia or near-work time may be reduced with increased time outdoors in children between 8 and 12 years. Moreover, some clinical trials [87,91,92,93] have evidenced that myopia incidence decreases in children between 6 and 11 years when the time spent outdoors is between 40 and 80 min per day. The Guangzhou Outdoor Activity Longitudinal Trial [92], which was a randomized clinical trial performed in children aged 6–7 years, obtained a reduction in myopia incidence from 39.5% to 30.4% when a 40 min daily class of outdoor activity was added over 3 years. Similarly, the ROCT711 program trial in Taiwan [93] another randomized clinical trial, found a 17% decrease in myopia incidence in children, aged 6–7 years, who spent outdoor time up to 11 h per week during 1 year. 

Regarding myopia progression, former studies in children (6–14 years) indicated no relationship of outdoor activity with myopia progression [66,94] nor myopia stabilization [70]. The Anyang Childhood Eye Study [69] in a sample of children from 10 to 15 years disclosed that a slower elongation rate was related to outdoor activity only in those children who were non-myopic at the study baseline. To all appearances, outdoor activity has shown to be a key factor in reducing myopia incidence but not in slowing its progression. Indeed, a meta-analysis [21] based on articles that involved samples aged from 6 to 18 years has recently reported the effect of protection on both myopia incidence and prevalence, but not on myopia progression. However, a 23-year follow-up study [95] found a slower myopic progression rate among myopic subjects who spent more than 3 h a day on outdoor activities. In the North India Myopia Study [96], it was also obtained that outdoor activity for longer than 2 h might be protective against myopia progression in children between 5 and 15 years. Conforming with this, the randomized trial of Wu et al. [93] obtained a reduction in myopia progression of 30% in 1 year by performing outdoor activity for up to 11 h per week in children aged 6 and 7 years. For now, further studies are needed to support the possible inhibition of myopia progression due to outdoor activity.

Several theories have emerged to explain the biological mechanism underlying the protective effect of outdoor activity, among which are the increase in light exposure, dopamine release, vitamin D, or the increased depth of field [97]. According to the light intensity hypothesis referred to by Rose et al. [85], slower axial elongation is associated with greater daily light exposure in the study of Read, Collins, and Vincent [98]. This last study reported that brighter light intensities above 3000 lux are required for greater influence on eye growth slowdown. In the same line, the ROCT711 program trial [93] stated that protection against myopia could be achieved with short periods of high light intensity or otherwise long periods of moderate light intensity. The light–dopamine theory proposes that higher light intensity mediates the release of the dopamine retinal transmitter [97], which has demonstrated a role in axial growth regulation [99]. Similarly, vitamin D theory supports that ultraviolet light stimulates vitamin D production, which has a relationship with axial growth and myopic pathogenesis [100,101]. Finally, the increased depth of focus theory is also related to light, since the depth of focus is known to increase with pupil constriction, and therefore, it would lead to a decrease in the retinal image blur [97].

#### 3.2.3. Life Environment

The residence in an urban or rural environment is also considered as a risk factor. Epidemiology studies in children (7–17 years) have shown that between populations with similar genetic ancestry, lower prevalence is found in those who have grown up in rural environments [44,102,103,104,105]. Significant differences in myopia progression have also been obtained in some studies in children between 5 and 16 years [106,107]. These differences may be attributable to educational and socioeconomic levels, which tend to be higher in the urban environment. Some authors [46,108] suggested that higher myopia prevalence is related to greater family income and education. Similarly, smaller house space has been associated with more myopia [43]. Indeed, a recent study [109] stated the defocus profile in the home environment as a potential myopia risk factor in children (7–12 years).

Myopia prevalence differences attributed to the life environment could also involve near work and time outdoors. However, population density has exhibited an association with greater myopia prevalence regardless of time outdoors and near work in Australian and Chinese children (7–15 years) [43,45]. Higher population density was related to longer AL and more negative refractive error in two studies on children (7–12 years) [110,111]. Recently, Read et al. [112] acquired on children (8–12 years) different outdoor light exposure between two urban locations in Australia and Singapore, respectively. Likewise, children (6–7 years) of Chinese ethnicity who grew up in Singapore have demonstrated to have differences in outdoor time activity compared to those age-matched in Sydney [113]. More studies are required to establish the mechanism that lies behind these associations.

#### 3.2.4. Digital Screen Time

As technology becomes an integral part of our daily lives, the use of digital devices has been suggested as a potential risk factor for myopia development. Some authors have revealed a relationship between digital screen time and myopia in children [57,67,114,115,116,117,118,119,120]. Two studies [114,117] carried out on Indian schoolchildren (5–15 years) pointed out that prolonged screen time led to an increased risk of myopia between 4- and 8-fold. Similarly, another two studies [115,116] reported that screen time was associated with higher myopia prevalence in Caucasian children (6–7 years; 16–17 years). Other authors [119,120] have acquired greater myopia error and longer axial length in children (8–9 years; 6–14 years) related to increased screen time.

On the contrary, several studies [24,27,62] have not found an association between screen time and myopia. So far, there is no consistent evidence to prove the association between screen time and myopia development. Indeed, the increase in myopia prevalence occurred long before the extended use of electronic devices [2]. Nonetheless, Dirani et al. [121] have suggested that device screen time is a risk factor that accounts for both increased near-work time and decreased time outdoors. Accordingly, myopic children (8–13 years) have been reported to spend more time indoors performing near-vision tasks and using digital screens at a proximal distance than non-myopic children [102,118]. However, further studies are needed to prove if prolonged screen time might have a substitute effect replacing traditional near-work tasks such as writing and reading.

**Table 2:** Myopia risk factors.

	Relationship	Main Findings	Related Factors
Parental myopia	Strong	Two myopic parents: ×5 odds * [26,27] One myopia parent: ×2 odds * [26,27]	Gene–environment interaction
Education	Strong	School length and the performance intensity associated [48,49,50,51]	Near work Accommodative lag
Near work	Moderate	Continuous reading (>30 min): ×1.5 odds * [62] Closer reading distance (<30 cm): ×2.5 odds * [62]	Education Accommodative lag
Time outdoors	Strong	Time outdoors between 40–80 min associated with reduced myopia incidence [87,91,92,93]	Light exposure, dopamine release, vitamin D, and increased depth of field
Light exposure	Moderate	Slower axial elongation is associated with greater daily light exposure (>3000 lux) [85,98]	Dopamine release, vitamin D, and increased depth of field
Life environment	Weak	Higher myopia prevalence in urban environments [44,102,103,104,105]	Education, near work, and time outdoors
Digital screen	Weak	Prolonged screen time: ×4–8 odds * [114,117]	Near work and time outdoors

* Risk of having myopia.

## 4. Discussion

It is important to note that myopia is a complex condition influenced by multiple factors, and individual susceptibility can vary. A combination of genetic predisposition, visual habits, and environmental factors likely contribute to the development of young-onset myopia. 

Family history plays a significant role in the development of myopia, as confirmed by familial and genome-wide association studies. Having myopic parents greatly increases the likelihood of a child developing myopia. Therefore, the prevalence of myopia rises with the number of myopic parents [23,24,25,26]. Nonetheless, the relationship between the refractive error of children and their parents is complex and multifaceted. While genetics undoubtedly play a crucial role, the shared environment within families, including visual habits and lifestyle factors, also contributes to the observed correlation. Genetic factors impose a level of baseline risk of myopia and might mediate the influence level of environmental risk factors [11].

Educational level strongly correlates with myopia prevalence, aligning with the fact that myopia tends to progress during school years. Higher education level was associated with higher myopia prevalence in several studies [6,8,10,46,47]. In some cultures, there is a strong emphasis on academic achievements, leading to intense academic competition and pressure on students to excel. Indeed, numerous studies [11,48,49,50,51] have demonstrated a strong correlation between educational intensity and myopia prevalence. Hence, the link between education and myopia is strong and consistent and shows up in the association of myopia with school performance [24,52] and length [51]. 

The relationship between education and myopia suggests that educational systems that require extensive near-work activities are associated with increased prevalence and severity of myopia [56,57,58,59,60,61,62]. However, studies in children have shown mixed results regarding the correlation between near work and myopia. Some studies suggest a positive association [57,58,59,60,61,62,63], while others do not report such association [25,27,64]. Prolonged and intense near work during childhood and adolescence has been associated with an increased risk of myopia development [59]. Concretely, continuous reading (>30 min) and closer reading distance (<30 cm) have been linked with a higher risk of myopia onset [62] and progression [61]. 

Limited exposure to natural outdoor light, especially during childhood, has been linked to an increased risk of myopia. Increased time spent outdoors between 40 and 80 min per day has been associated with reduced myopia incidence in several clinical trials carried out in children between 6 and 11 years [87,91,92,93]. Therefore, time outdoors, especially in bright sunlight, has been suggested to have a protective effect against myopia development [85]. The intensity and duration of light exposure play a crucial role in influencing eye growth. Bright outdoor light helps regulate eye growth and may help prevent myopia progression. [85,98]. Moreover, residing in urban versus rural environments has been considered a risk factor, perhaps due to the interplay of educational and lifestyle factors in urban populations. Populations with similar genetic backgrounds but different living environments exhibit varying myopia prevalence, often attributed to educational and socioeconomic factors [46,108]. 

With technological advances, children and young adults are spending an increasing amount of time using electronic devices such as smartphones, tablets, and computers. Besides, students are exposed to electronic devices more than ever before because of educational digitalization. As explained above, spending excessive time engaged in near-work activities such as reading or using digital devices may contribute to myopia development. This is often referred to as the “near work” hypothesis [20]. Consequently, the use of digital devices has been proposed as a potential risk factor for myopia development. Some studies have reported a relationship between increased screen time and myopia [57,67,114,115,116,117,118,119,120]; meanwhile, other authors have not found this correlation [24,27,62]. The association between screen time and myopia might be confounded by factors such as increased near-work time and reduced outdoor time. Some studies [102,118] found that myopic children tend to spend more time indoors with digital screens, which led to a decreased time outdoors. Nevertheless, more research is needed to understand the full impact of screen time on myopia development.

Finally, early interventions, such as spending time outdoors, taking regular breaks during near-work tasks, and receiving regular eye check-ups, can help mitigate the risk of myopia development in children and young individuals. Understanding both genetic and environmental influences is important for developing strategies to manage and prevent myopia in children. Future directions in this field are primarily focused on taking proactive measures to address myopia. At a public health level, interventions within schools to increase outdoor time for children can immediately mitigate myopia onset and subsequently reduce high myopia prevalence. Revising curricula and school systems to alleviate educational pressures, thus allowing for more outdoor time, is another avenue worth exploring. Objective measurement techniques should be employed to better understand children’s activity patterns, aiding in the precise definition of protective exposures.

## 5. Conclusions

In summary, myopia development and progression have been shown to be related to gene–environment interaction. Despite the direct role of genetic contribution in myopia incidence, some genotypes are more susceptible to changes than others in the same environment. Identifying risk factors and underlying processes in this context is essential for guiding clinical practices. Educational intensity is one of the environmental factors associated with both myopia development and progression. Near-work tasks are considered involved in such association, especially when performed for a prolonged time (>30 min) and with reduced distance (<30 cm). Further, the increased time spent outdoors has been suggested as a protective factor from myopia development. However, up to now, there are not enough studies to evidence the possible inhibition of myopia progression due to outdoor activity. Therefore, the interventional strategy suggested so far is mainly the increased time outdoors (40–80 min per day). This might be combined with the reduction in the time spent doing near-work tasks, although its effectiveness has not been proven in clinical trials so far. It is important to acknowledge the progress made thus far, as actionable steps can now be taken at both public health and clinical levels to attenuate myopia and high myopia development. 

## Figures and Tables

**Table 1:** Eligibility criteria.

	Inclusion Criteria	Exclusion Criteria
Study types	Articles, reviews, systematic reviews, and clinical trials	Studies with animals, case reports or books
Publication year	January 2000–May 2023	Before 2000
Age	Children and teenagers 5–30 years *	Adults Older than 30 years
Ethnicity	Caucasian and Asian	Others
Language	English	Other languages
Accessibility	Full-text available online	Full-text non-available online

* Except for genetics articles that are only carried out in adults.

## Data Availability

Not applicable.
